# The merit of proton magnetic resonance spectroscopy in the longitudinal assessment of spinocerebellar ataxias and multiple system atrophy-cerebellar type

**DOI:** 10.1186/s40673-014-0017-4

**Published:** 2014-12-01

**Authors:** Hung-Chieh Chen, Jiing-Feng Lirng, Bing-Wen Soong, Wan Yuo Guo, Hsiu-Mei Wu, Clayton Chi-Chang Chen, Cheng-Yen Chang

**Affiliations:** Department of Radiology, National Yang-Ming University School of Medicine, Taipei, Taiwan; Department of Radiology, Taichung Veterans General Hospital, Taichung, Taiwan; Department of Radiology, Taipei Veterans General Hospital, Taipei, Taiwan; Department of Neurology, National Yang-Ming University School of Medicine and Taipei Veterans General Hospital, 155, Sec. 2, Linung St, Taipei, Taiwan; Department of Neurology, Taipei Veterans General Hospital, Taipei, Taiwan

**Keywords:** Proton MRS, SARA, Spinocerebellar ataxias, Multiple system atrophy- cerebellar type

## Abstract

**Background:**

Spinocerebellar ataxia (SCA) and multiple system atrophy-cerebellar type (MSA-C) often present with similar clinical manifestations in the beginning. Magnetic resonance spectroscopy (MRS) has been proved to be a useful tool to help differentiate different types of SCA and MSA-C on cross-sectional studies. However, longitudinal changes of the MRS metabolites in these subjects have never been reported. The purpose of this study was to track the longitudinal evolution of the MRS metabolites in these patients and to ascertain the correlation between clinical severity measured by Scale of the Assessment and Rating of Ataxia (SARA) and MRS metabolites.

**Results:**

Significant reductions of NAA/Cr and NAA/Cho in the cerebellar hemispheres in all patients and lower Cho/Cr in the cerebellar hemispheres in patients with SCA2 or MSA-C were found at all times. At initial assessments, patients with MSA-C or SCA2 tended to have lower NAA/Cr and Cho/Cr in the cerebellar hemispheres than those with SCA3 or SCA6. At follow-ups, patients with SCA2 or MSA-C had a lower NAA/Cr in cerebellar hemispheres than those with SCA3 or SCA6. Patients with MSA-C had a lower NAA/Cr in the vermis and Cho/Cr in the cerebellar hemispheres than those with SCA2 at the start, and had a lower NAA/Cr in cerebellar hemispheres than those with SCA2 at follow-ups.

**Conclusion:**

Characteristic patterns of neurodegenerative evolution were observed in patients with disparate SCAs and MSA-C using MRS and SARA. A continual impairment of neuronal integrity was observed in all groups of patients. The longitudinal changes of MRS metabolites and SARA scores were most striking in patients with SCA2 and MSA-C. Although the changes in the metabolites on MRS may still be used to help understand the pathophysiology of ataxia disorders, they are short of being a good biomarker.

## Background

Spinocerebellar ataxia (SCA) is the most common ataxia syndrome inherited in an autosomally dominant manner and may be further categorized into 35 genotypes [1]. Multiple system atrophy-cerebellar type (MSA-C) is the most common sporadic cerebellar ataxia with a rapidly progressive course. The diagnosis of MSA-C is made according to the clinical features fitting the international consensus statement [2]*.* Both types of ataxia disorders present with similar clinical manifestations initially. An early and precise diagnosis is important in that it not only foretells the prognosis, allows for comprehensive genetic counseling, but also helps design the best clinical management for each patient. Scale for the Assessment and Rating of Ataxia (SARA) [3] has been validated to be a reliable semi-quantitative clinical scale of ataxia which satisfies accepted criteria of reliability and can be used to measure the clinical severity and progression of diseases with ataxia.

Magnetic resonance spectroscopy (MRS) provides a measure of brain chemistry and has been widely used to non-invasively evaluate *in vivo* changes of the metabolites in the brain. Creatine (Cr) provides a measure of energy stores in the brain. It is very stable and is usually used as a reference for comparison. N-acetylaspartate (NAA) is a marker for neuronal integrity or volume [4,5] and a reduction of NAA denotes a pathological process that adversely affects neuronal integrity [6–9]. Choline (Cho) is a measure of cellular turnover, reflecting the content of cytosolic glycerolphosphocholine and phosphocholine and representing the products of membrane phosphotidylcholine breakdown, precursors of choline and acetylcholine synthesis [10]. A reduction in Cho reflects impairment in the production of cell membranes, the precursor of neurotransmitter acetylcholine and acetylcholine itself. NAA/Cho has been used as a marker for cerebral metabolism [6,11,12]*.*

In the literature, there have been several cross-sectional studies on the changes of MRS metabolites in patients with SCA or MSA-C [13–18], including one from our group reporting the differences of MRS metabolites between SCAs and MSA-C [18]. MRS has also been used to longitudinally follow the progression of other central nervous system disorders, such as dementia [19], mania [20], traumatic brain injury [21] and HIV encephalopathies [22]. The changes of metabolites in MRS faithfully correlated with the changes in clinical severity.

In this paper, we compare the longitudinal changes of MRS metabolites in the brain and SARA scores in patients with disparate types of SCAs and MSA-C and ascertain their correlations.

## Results

The patients with SCA3 were younger than those with SCA2 and MSA-C (Table 1). Otherwise, there was no significant difference between patient groups in terms of disease duration, SARA scores at the time when the first or the second MRS assessment was performed, or the time interval in between two assessments.

**Table 1:** **Demographic features of the subjects**

			**First MRS assessment**		**Second MRS assessment**		
	**Number of patients**	**Age (years)**	**Disease duration (months)**	**SARA scores**	**Disease duration (months)**	**SARA scores**	**Interval in between 2 MRS assessments (months)**
*p* ^#^		0.03	0.326	0.195	0.736	0.114	0.680
SCA2	5	60.6 ± 11.1^*^	3.7 ± 3.03	8.4 ± 3.13	43.0 ± 15.47	17.13 ± 7.1	38.63 ± 13.21
SCA3	18	46.3 ± 10.0^*^	6.14 ± 3.96	10.97 ± 6.28	49.91 ± 23.82	15.28 ± 5.23	43.78 ± 21.74
SCA6	3	59.0 ± 13.9	6.83 ± 2.36	12.5 ± 2.65	42.25 ± 15.2	19.0 ± 1.41	34.5 ± 17.68
MSA-C	12	60.6 ± 6.1^*^	4.25 ± 2.20	15.29 ± 6.71	38.7 ± 12.33	22.35 ± 7.02	34.3 ± 12.82
HC	44	51.1 ± 17.9	--	--	--		

*SCA2*: Spinocerebellar ataxia type 2; *SCA3*: Spinocerebellar ataxia type 3; *SCA6*: Spinocerebellar ataxia type 6; *MSA*: Multiple system atrophy- cerebellar type; *HC*: Healthy controls.

^*^The patients with SCA 3 were significantly younger than those with SCA2 (*p* = 0.014) or MSA-C (*p* = 0.001).

^#^Kruskal-Wallis test.

**Figure 1:**
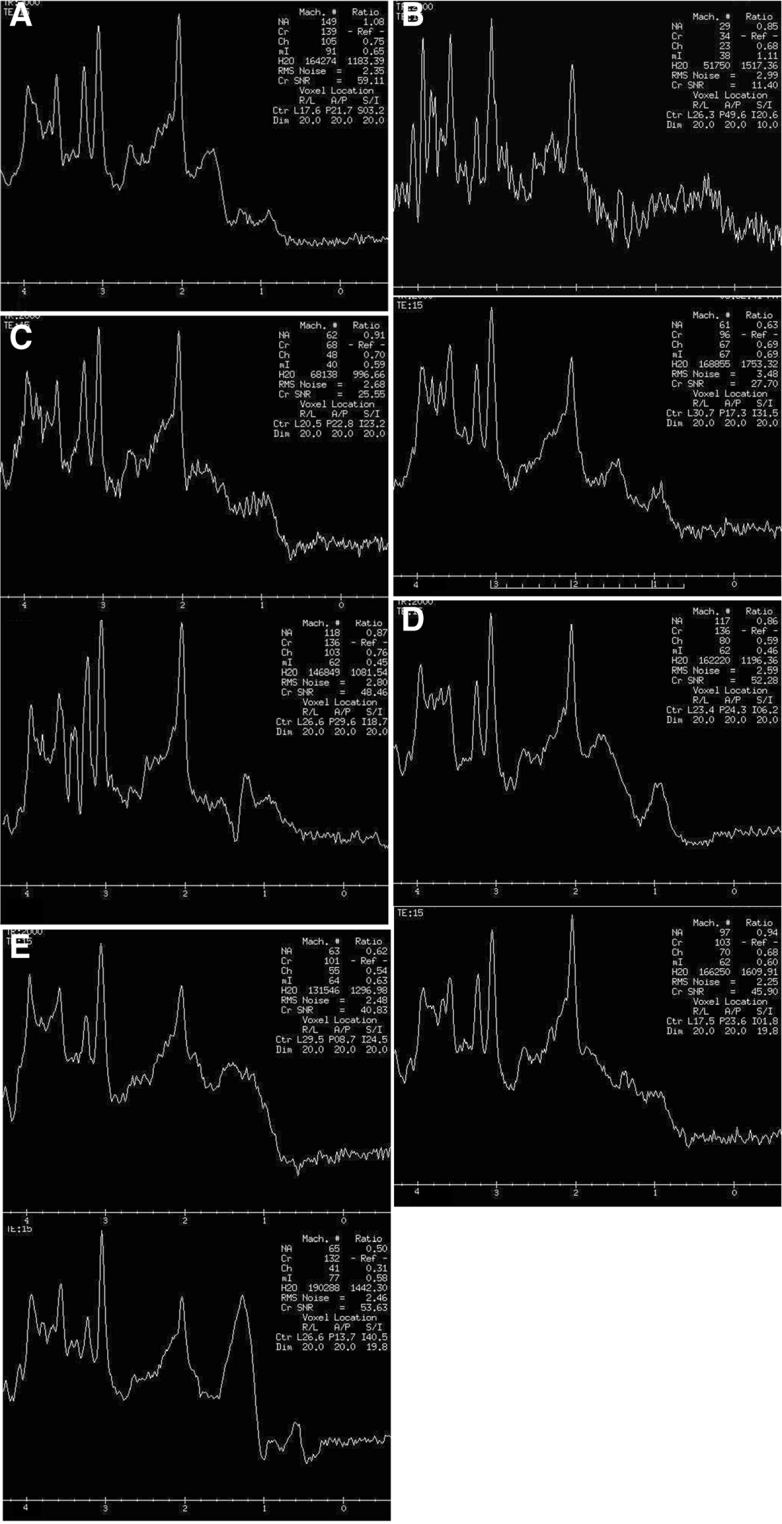
**The MR spectroscopy of the patients at the initial (upper) and follow-up (lower) assessments. (A)** healthy controls, **(B)** SCA2, **(C)** SCA3, **(D)** SCA6 and **(E)** MSA-C.

The MRS of patients with different SCAs and MSA-C were demonstrated in Figure 1.

### Patients with MSA-C

In patients with MSA-C, the NAA/Cr, Cho/Cr and NAA/Cho in both the cerebellar hemispheres and vermis were significantly reduced (*p* < 0.001) at both MRS assessments, as compared with those of the healthy controls (Table 2).

**Table 2:** **Comparison of** ^**1**^ **H MRS metabolites between the patients with SCA, MSA-C and controls and between 2 assessments in each group**

		**Metabolites on**		**The 1** ^**st**^ **MRS** ^*^ **vs. controls**	**The 2** ^**nd**^ **MRS** ^*^ **vs. controls**	**1** ^**st**^ **vs. 2** ^**nd**^ **MRS** ^**#**^
		**the 1** ^**st**^ **MRS**	**the 2** ^**nd**^ **MRS**			
**Cerebellar hemispheres**	**NAA/Cr**					
	SCA2	0.65 ± 0.10	0.72 ± 0.06	0.000	0.000	0.113
	SCA3	0.88 ± 0.10	0.85 ± 0.08	0.000	0.000	0.073
	SCA6	0.83 ± 0.07	0.87 ± 0.08	0.001	0.014	0.440
	MSA-C	0.58 ± 0.14	0.62 ± 0.23	0.000	0.000	0.331
	HC	1.00 ± 0.12	-	-	-	-
	**Cho/Cr**					
	SCA2	0.58 ± 0.07	0.62 ± 0.05	0.001	0.019	0.031
	SCA3	0.68 ± 0.09	0.65 ± 0.08	0.392	0.027	0.098
	SCA6	0.69 ± 0.08	0.65 ± 0.04	0.710	0.279	0.116
	MSA-C	0.50 ± 0.10	0.52 ± 0.14	0.000	0.000	0.955
	HC	0.70 ± 0.09	-	-	-	-
	**NAA/Cho**					
	SCA2	1.14 ± 0.27	1.16 ± 0.16	0.004	0.000	0.796
	SCA3	1.30 ± 0.17	1.31 ± 0.15	0.000	0.001	0.768
	SCA6	1.23 ± 0.15	1.34 ± 0.10	0.008	0.174	0.113
	MSA-C	1.17 ± 0.29	1.21 ± 0.28	0.000	0.000	0.263
	HC	1.45 ± 0.19	-			
**Vermis**	**NAA/Cr**					
	SCA2	0.74 ± 0.05	0.70 ± 0.09	0.000	0.003	0.421
	SCA3	0.82 ± 0.07	0.80 ± 0.08	0.002	0.000	0.205
	SCA6	0.79 ± 0.06	0.76 ± 0.06	0.024	0.043	0.126
	MSA-C	0.63 ± 0.08	0.64 ± 0.08	0.000	0.000	0.966
	HC	0.90 ± 0.11	-	-	-	-
	**Cho/Cr**					
	SCA2	0.61 ± 0.08	0.55 ± 0.06	0.062	0.003	0.233
	SCA3	0.66 ± 0.07	0.65 ± 0. 08	0.433	0.194	0.910
	SCA6	0.70 ± 0.03	0.65 ± 0.06	0.494	0.463	0.451
	MSA-C	0.54 ± 0.06	0.50 ± 0.11	0.000	0.000	0.178
	HC	0.68 ± 0.07	-	-	-	-
	**NAA/Cho**					
	SCA2	1.22 ± 0.19	1.28 ± 0.07	0.174	0.380	0.833
	SCA3	1.25 ± 0.13	1.22 ± 0.13	0.072	0.002	0.170
	SCA6	1.13 ± 0.10	1.17 ± 0.02	0.024	0.065	0.766
	MSA-C	1.16 ± 0.08	1.31 ± 0.24	0.000	0.991	0.083
	HC	1.32 ± 0.18	-			

*SCA2*: Spinocerebellar ataxia type 2; *SCA3*: Spinocerebellar ataxia type 3; *SCA6*: Spinocerebellar ataxia type 6; *MSA-C*: Multiple system atrophy-cerebellar type; *HC*: Healthy controls.

^*^Mann–Whitney test, *p* value.

^#^Pair-t test, *p* value.

### Patients with SCA

In all types of SCAs studied, the NAA/Cr in the cerebellar hemispheres and vermis and NAA/Cho in the cerebellar hemispheres were significantly reduced (*p* < 0.05) at both assessments (Table 2).

In patients with SCA2, the Cho/Cr in the cerebellar hemispheres was significantly reduced (*p* < 0.05) at both MRS assessments (Table 2) and in the vermis only at the second MRS assessment, as compared with the healthy controls.

In patients with SCA3, NAA/Cho in the vermis appeared consistently reduced and Cho/Cr in the cerebellar hemisphere became significantly reduced than healthy controls at 2^nd^ MRS assessment (*p* < 0.05). (Table 2) In patients with SCA6, NAA/Cho appeared significantly reduced in the vermis at initial assessment. (*p* = 0.024) (Table 2).

#### Comparison between patients with different types of SCA and MSA-C

At the first MRS assessment (Table 3), patients with MSA-C had a significantly lower NAA/Cr and Cho/Cr in the cerebellar hemispheres and vermis than those with SCA3 or SCA6, and a lower NAA/Cho in the cerebellar hemispheres than those with SCA3. Furthermore, patients with SCA2 had a significantly lower NAA/Cr and Cho/Cr in the cerebellar hemispheres than those with SCA3 or SCA6.

**Table 3:** **The differences in MRS metabolites between patient groups at the 1** ^**st**^ **MRS assessment**

		**Disease**	**SCA3** ^*^	**SCA6** ^*^	**MSA-C** ^*^
Cerebellar hemispheres	**NAA/Cr**	SCA2	0.000	0.004	0.155
		SCA3	-	0.356	0.000
		SCA6	-	-	0.000
	**Cho/Cr**	SCA2	0.010	0.039	0.035
		SCA3	-	0.985	0.000
		SCA6	-	-	0.002
	**NAA/Cho**	SCA2	0.236	0.606	0.743
		SCA3	-	0.356	0.018
		SCA6	-	-	0.401
Vermis	**NAA/Cr**	SCA2	0.037	0.297	0.013
		SCA3	-	0.487	0.000
		SCA6	-	-	0.024
	**Cho/Cr**	SCA2	0.256	0.180	0.064
		SCA3	-	0.252	0.001
		SCA6	-	-	0.011
	**NAA/Cho**	SCA2	0.730	0.456	0.493
		SCA3	-	0.158	0.092
		SCA6	-	-	0.665

*SCA2*: Spinocerebellar ataxia type 2; *SCA3*: Spinocerebellar ataxia type 3; *SCA6*: Spinocerebellar ataxia type 6; *MSA-C*: Multiple system atrophy- cerebellar type.

*Mann–Whitney test, *p*-value.

At the second MRS assessment (Table 4), patients with MSA-C still had lower NAA/Cr, Cho/Cr in the cerebellar hemispheres and vermis than those with SCA3 and lower NAA/Cr in the cerebellar hemispheres than those with SCA2 and SCA6. Patients with SCA2 continued to have lower NAA/Cr in the cerebellar hemispheres than those with SCA3 or SCA6.

**Table 4:** **The differences in MRS metabolites between patient groups at 2** ^**nd**^ **MRS assessment**

		**Disease**	SCA3*	SCA6*	MSA-C*
Cerebellar hemispheres	NAA/Cr	SCA2	0.001	0.014	0.017
		SCA3	-	0.538	0.000
		SCA6	-	-	0.022
	Cho/Cr	SCA2	**0.197**	**0.393**	**0.055**
		SCA3	**-**	**0.979**	**0.001**
		SCA6	**-**	**-**	**0.067**
	**NAA/Cho**	SCA2	**0.026**	**0.089**	**0.617**
		SCA3	**-**	**0.456**	**0.077**
		SCA6	**-**	**-**	**0.202**
Vermis	**NAA/Cr**	SCA2	**0.064**	**0.355**	**0.356**
		SCA3	**-**	**0.526**	**0.000**
		SCA6	**-**	**-**	**0.085**
	**Cho/Cr**	SCA2	**0.017**	**0.064**	**0.480**
		SCA3	**-**	**0.880**	**0.003**
		SCA6	**-**	**-**	**0.133**
	**NAA/Cho**	SCA2	**0.484**	**0.165**	**0.777**
		SCA3	**-**	**0.233**	**0.202**
		SCA6	**-**	**-**	**0.390**

*SCA2*: Spinocerebellar ataxia type 2; *SCA3*: Spinocerebellar ataxia type 3; *SCA6*: Spinocerebellar ataxia type 6; *MSA-C*: Multiple system atrophy-cerebellar type.

*Mann–Whitney test, *p* value.

#### *Longitudinal changes in the MRS* metabolites

**Figure 2:**
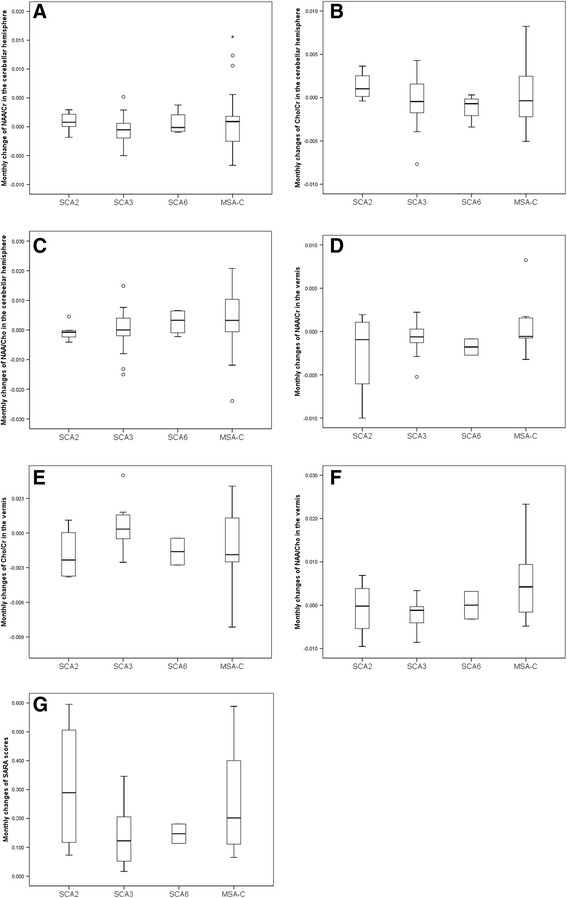
**Monthly changes of the MRS metabolites and the SARA scores. (A)** NAA/Cr in the cerebellar hemispheres, **(B)** Cho/Cr in the cerebellar hemispheres, **(C)** NAA/Cho in the cerebellar hemispheres, **(D)** NAA/Cr in the vermis, **(E)** Cho/Cr in the vermis, **(F)** NAA/Cho in the vermis. **(G)** Changes of total SARA scores.

There was no significant difference in the MRS metabolites between initial and follow up assessments in the study groups (Table 2), except for an increase of Cho/Cr with time in the cerebellar hemispheres in patients with SCA2 (*p* = 0.031) (Table 2). Furthermore, there was no difference in the monthly changes of metabolites between different groups of patients (Table 5 and Figure 2).

**Table 5:** **The monthly changes of MRS metabolites in different groups of patients** ^╥^

	**Cerebellar hemispheres**			**Vermis**		
	**NAA/Cr**	**Cho/Cr**	**NAA/Cho**	**NAA/Cr**	**Cho/Cr**	**NAA/Cho**
*p* ^#^	0.471	0.168	0.261	0.693	0.269	0.273
SCA2	0.09 ± 0.17	0.13 ± 0.14	−0.08 ± 0.28	−0.25 ± 0.53	−0.18 ± 0.23	−0.08 ± 0.68
SCA3	−0.04 ± 0.22	−0.04 ± 0.26	0.01 ± 0.61	−0.07 ± 0.20	0.05 ± 0.21	−0.22 ± 0.37
SCA6	0.06 ± 0.22	−0.11 ± 0.16	0.27 ± 0.44	−0.18 ± 0.13	−0.16 ± 0.16	0.00 ± 0.45
MSA-C	0.14 ± 0.59	0.02 ± 0.37	0.33 ± 1.14	0.03 ± 0.32	−0.14 ± 0.36	0.51 ± 0.86

*SCA2*: Spinocerebellar ataxia type 2; *SCA3*: Spinocerebellar ataxia type 3; *SCA6*: Spinocerebellar ataxia type 6; *MSA-C*: Multiple system atrophy- cerebellar type.

^╥^Data are presented as 100X MRS ratios.

^#^Kruskal-Wallis test.

#### Longitudinal changes in the SARA scores

There were significant increases of SARA scores with time in patients with SCA2 (*p* = 0.031), SCA3 (*p* < 0.001) and MSA-C (*p* < 0.001), but not in SCA6 (*p* = 0.318) (Table 6). Moreover, if we compared the changes of SARA on a monthly basis, patients with MSA-C and SCA2 stood out and seemed to have a faster progression than those with SCA3 or SCA6 (Table 6, Figure 2G), although the difference had not reached a statistical significance.

**Table 6:** **The monthly changes of SARA scores in different groups of patients**

	**SARA score at the 1** ^**st**^ **assessment**	**SARA score at the 2** ^**nd**^ **assessment**	**1** ^**st**^ **vs. 2** ^**nd**^ **SARA scores** ^**@**^	**Monthly changes of SARA**
*p* ^#^	0.195	0.114		0.343
SAC2	8.4 ± 3.13	17.13 ± 7.1	0.031	0.31 ± 0.24
SAC3	10.97 ± 6.28	15.28 ± 5.23	0.000	0.15 ± 0.10
SAC6	12.5 ± 2.65	19.0 ± 1.41	0.318	0.15 ± 0.05
MSA-C	15.29 ± 6.71	22.35 ± 7.02	0.000	0.26 ± 0.18

^#^Kruskal-Wallis test.

^@^Pair-t test, *p* value.

#### The correlation between the changes of MRS metabolites and SARA scores

**Figure 3:**
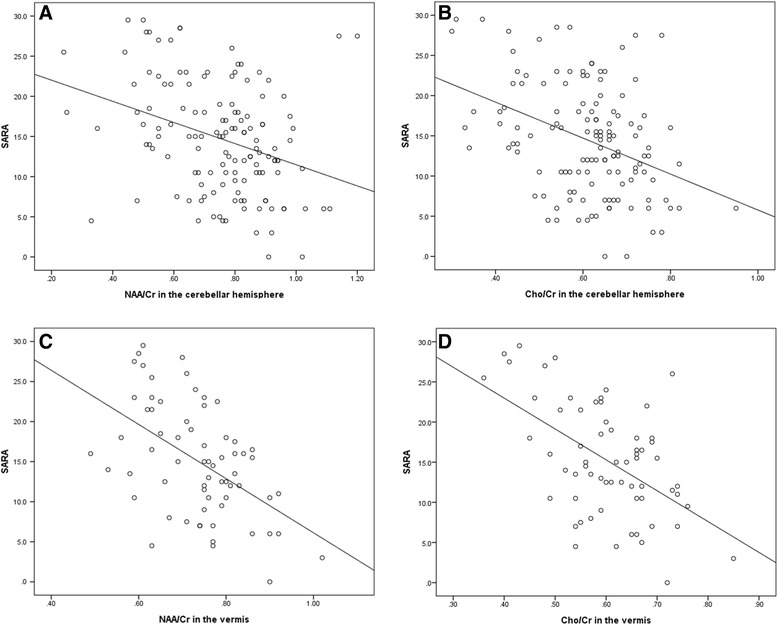
**The correlation between SARA and MRS metabolites. (A)** NAA/Cr in the cerebellar hemispheres vs. SARA, **(B)** Cho/Cr in the cerebellar hemispheres vs. SARA, **(C)** NAA/Cr in the vermis vs. SARA and **(D)** Cho/Cr in the vermis vs. SARA.

The changes of NAA/Cr (R = −0.332, *P* < 0.001 in the cerebellar hemispheres; R = −0.521, *P* < 0.001 in the vermis) and Cho/Cr (R = −0.380; *P* < 0.001 in the cerebellar hemispheres; R = −0.529, *P* < 0.001 in the vermis) were inversely correlated with the changes of SARA scores (Figure 3).

## Discussion

To our knowledge, this is the first study designed to evaluate the longitudinal changes of metabolites on MRS in conjunction with the clinical severity measured with SARA in patients with ataxia syndromes. Our study has several strengths. First, the same MR instrument and study protocol were used throughout. Second, the clinical severity was evaluated uniformly by a single board-certified neurologist who is experienced in evaluating ataxia with the same valid assessment scale, SARA. Third, the disease duration and clinical severity at two assessments were similar across different diseases.

In this study, a continual impairment in neuronal integrity, as indicated by the reduction in NAA/Cr in the cerebellar hemispheres and vermis (Table 2), at both initial and follow-up assessments was found in all groups of patients. Over the timespan of 2–4 years, not much difference in MRS metabolites was observed between two MRS assessments (Table 2) in all SCA subgroups and MSA-C, suggesting that MRS metabolites are not sensitive enough markers to track the changes in cerebellar metabolism over the limited time span of follow-up (Table 2). Further study with larger sample sizes might be needed to validate these observations. On the other hand, SARA scores faithfully reflected the deterioration in severity of SCA2, SCA3 and MSA-C over the time span (Table 6), implicating that SARA qualifies as a more sensitive marker to gauge the progression of cerebellar ataxias. Nevertheless, the changes of NAA/Cr and Cho/Cr in the cerebellar hemispheres and vermis somehow still inversely correlated with the changes in SARA scores. The evolution of different metabolites on MRS could still, to a certain degree, help understand the pathophysiology of the disease.

### MSA-C

In general, patients with MSA-C tended to have a worse neuronal integrity and less production of cell membranes than those with SCA3 or SCA6 at the first MRS assessment (Tables 2 & 3). With time, the differences remained similar at follow-ups (Table 4), suggesting a persistent and more severe disruption in the neuronal and membrane integrity in MSA-C. MSA-C is a rapidly progressive neurodegenerative disease manifesting ataxia and autonomic dysfunction [23]. Our previous cross-sectional study in patients with MSA-C also corroborated these observations [18].

MSA-C and SCA2 are two relentlessly and rapidly progressive diseases manifesting ataxia with neuronal drop-outs and impairment of membrane synthesis. Lactate peak and myo-inositol were previously proposed to be indicators to differentiate SCA2 from MSA-C [14–16]. In this study, we found that, at the early stage of the diseases, the MRS features were similar between MSA-C and SCA2. As disease progressed, the different speed of changes in Cho/Cr and NAA/Cr in the cerebellar hemispheres might help differentiate MSA-C from SCA2 (Table 4).

### SCA

In line with previous reports [15,16], patients with SCA2 have a more severe impairment in neuronal integrity, given the lower NAA/Cr in the cerebellar hemispheres than those with SCA3 or SCA6 at the start and at the follow-up assessment, even being of similar clinical severity (Tables 2, 3 & 4). The SARA scores increased significantly in SCA2 and SCA3 but not in SCA6 during the period of follow up (Tables 1 and 6). Although there was little difference in the monthly changes of SARA scores between subtypes of SCAs, the change was still higher in SCA2 (Table 6). Pathological studies in the past have revealed, in SCA2, a marked loss of Purkinje cells in the cerebellar cortex and a loss of myelinated fibers in the cerebellar peduncles and white matter, sparing the dentate nucleus, and the neuronal degeneration preceded the onset of clinical symptoms [24] .While a prominent spinopontine degeneration with a relative sparing of the olivocerebellar regions in SCA3 [25] and a pure cerebellar degeneration with a loss of Purkinje cells in the cerebellar cortex in SCA6 [26,27] were observed*.* The synaptic loss in the cerebellum and brainstem is most severe in SCA2 [28,29]. The MRS changes observed by us are corroborated by the pathological changes.

The disease progression reflected by the monthly changes of SARA scores was most severe in SCA2, MSA-C, followed by SCA3 and SCA 6. Similar results have been documented in our earlier report [30].

### Interval changes

Progressive reduction in the cerebellar volume, correlating with disease duration and age, has been documented in patients with MSA-C [31], SCA2 [32], SCA3 [33] and SCA6 [34]. In SCA2, the presence of pre-symptomatic region-specific atrophy was observed and a possible developmental component to the pathomechanisms was once proposed [32]. This is consistent with the early MRS changes in SCA2 when the clinical severity represented by SARA scores is still low and disease duration remains short.

The limitations of this study are, first, the sizes of the patients were relatively small, especially in SCA2 and SCA6. The small patient numbers could be the reason contributing to the lack of significance in disease duration between groups. We tried to minimize these heterogeneities by recruiting only patients who had their first MRS examination at a disease duration less than 12 months. Furthermore, the MRS metabolites from both cerebellar hemispheres were averaged for the ease of analyses, thus the data of MRS from the cerebellar hemispheres should actually be twice as many as those from the vermis. Hence, the reason that some differences of MRS in patient groups were observed only in the cerebellar hemispheres, but not in the vermis, could likely be attributed to the limited patient numbers. Third, we only focused on the metabolites in the cerebellar hemispheres and vermis. Fourth, we only correlated the metabolites on MRS with total SARA scores. The rates of progression in different compartments of SARA might be different [30]. Further studies with MRS in a larger cohort, addressing also the changes of metabolites in the other regions of the brain while correlating the changes between MRS metabolites and different compartments of SARA, would be desirable to help unravel the pathophysiology of the SCAs and MSA-C.

## Conclusion

Different patterns of neurodegeneration with impairment in neuronal integrity and defective membrane activities were observed in patients with SCAs and MSA-C in this longitudinal study. Although the changes on MRS negatively correlated with clinical severity (Figure 2), MRS does not seem to be a sensitive marker to assess the progress of ataxia syndromes. Nevertheless, the longitudinal changes of MRS and SARA scores in SCA2, SCA3 and MSA-C were different, both in the beginning and on the follow-up’s. Changes in MRS metabolites may still be used to help understand the underlying pathophysiology of ataxia disorders.

## Methods

### Patients and controls

This study was approved by the Institutional Review Board of Taipei Veterans General Hospital, Taipei, Taiwan. Patients who had undergone SARA and MR spectroscopy at least twice over a period of 10 years were enlisted. The interval between evaluations with SARA and the MR spectroscopy must be approximately within 3 months. From March 2004 to January 2014, a total of 47 patients with either one of the SCAs or MSA-C were studied sequentially in the afore-mentioned manner. Among them, 38 patients had their first MRS assessment with a disease duration less than 12 months and the follow-up MRS after one year. Among them, 5 were molecularly identified to be SCA2, 18 SCA3, and 3 SCA6. Twelve patients met the criteria of MSA-C. Forty-four healthy individuals without any history of neurological diseases served as controls. The MRS features in the basal ganglion were validated according to the normal data proposed by Brain Ross and Else Rubaek Danielsen [35]. The demographic features of the study subjects are listed in Table 1.

### Images and spectroscopic acquisition

Brain MRI and MRS were performed using a 1.5-T system (Signa EXCITE, GE Medical Systems, Milwaukee, WI). The MRI protocol consisted of an axial T1-weighted three-dimensional fast-spoiled gradient recalled acquisition in steady state images (TR 8.58 msec, TE 3.62 msec, inversion time [TI] 400 msec, voxel resolution 0.75X0.75X1.5 mm^3^) and an axial T2 fast spin-echo sequence [TR 4000 msec, TE 256.5 msec, voxel resolution 348X512].

After MR imaging, proton MRS was recorded in the cerebellar hemispheres and vermis by using single-voxel stimulated echo acquisition mode sequence (3000/15/13.7/96 [TR/TE/mixing time/excitations], spectral width = 2500 Hz, number of points = 2048, voxel resolution = 2 cm × 2 cm × 2 cm). The voxel of interest (VOI) in each subject was placed in a uniform manner by the same investigator (JFL). Care was taken to avoid cerebrospinal fluid space within the VOIs. The peak areas for N-acetyl aspartate (NAA) at 2.02 parts per million (ppm), Creatine (Cr) at 3.03 ppm, and Choline (Cho) at 3.22 ppm were measured using the Functool provided by the MR company (GE XVi, Milwaukee, WI). Peak integral values were expressed relative to the Cr peak. Metabolite intensity ratios were automatically calculated at the end of each single voxel acquisition including NAA/Cr and Cho/Cr. The NAA/Cho ratio was also calculated for comparison. MRS results with full width at half maximum (FWHM) > 6 Hz were disqualified from the MRS analyses to ensure high quality.

### Statistical analyses

Comparisons of the age at examination between the patient groups and healthy controls and comparison of the disease duration and the SARA scores at first and second evaluations between the patient groups were performed using nonparametric analyses (Kruskal-Wallis H test) given that some of the sample sizes were small and the assumption of a Gaussian distribution was not appropriate. Similarly, given the non-Gaussian distribution of the MRS parameters, comparison of the metabolites on MRS (NAA/Cr, Cho/Cr and NAA/Cho in the cerebellar hemispheres or vermis) between different groups of patients and healthy controls, between patients of disparate types of SCA and MSA-C, between patients with different types of SCA at initial and follow-up MRS assessments, and comparison of the monthly changes of metabolites on MRS and SARA scores were all performed using the non-parametric Mann–Whitney U-test. The interval changes of metabolites on MRS and SARA scores were evaluated by pair-t test. The correlation between metabolites on MRS and SARA scores was assessed with Pearson’s correlation coefficient. Differences were considered significant at *p <*0.05. Data are given as mean ± standard deviation.

## Abbreviations

SCA: Spinocerebellar ataxia
MSA-C: Multiple system atrophy-cerebellar type
MRS: Magnetic resonance spectroscopy
SARA: Scale of the Assessment and Rating of Ataxia
Cr: Creatine
NAA: N-acetylaspartate
Cho: Choline
VOI: Voxel of interest
